# Specificity of Motor Contributions to Auditory Statistical Learning

**DOI:** 10.5334/joc.351

**Published:** 2024-02-16

**Authors:** Sam Boeve, Riikka Möttönen, Eleonore H. M. Smalle

**Affiliations:** 1 Department of Experimental Psychology, Ghent University, Ghent, Belgium; 2 Cognitive Science, Department of Digital Humanities, University of Helsinki, Helsinki, Finland; 3 Department of Developmental Psychology, Tilburg University, Tilburg, Netherlands

**Keywords:** auditory statistical language learning, speech motor system, domain-specificity

## Abstract

Statistical learning is the ability to extract patterned information from continuous sensory signals. Recent evidence suggests that auditory-motor mechanisms play an important role in auditory statistical learning from speech signals. The question remains whether auditory-motor mechanisms support such learning generally or in a domain-specific manner. In Experiment 1, we tested the specificity of motor processes contributing to learning patterns from speech sequences. Participants either whispered or clapped their hands while listening to structured speech. In Experiment 2, we focused on auditory specificity, testing whether whispering equally affects learning patterns from speech and non-speech sequences. Finally, in Experiment 3, we examined whether learning patterns from speech and non-speech sequences are correlated. Whispering had a stronger effect than clapping on learning patterns from speech sequences in Experiment 1. Moreover, whispering impaired statistical learning more strongly from speech than non-speech sequences in Experiment 2. Interestingly, while participants in the non-speech tasks spontaneously synchronized their motor movements with the auditory stream more than participants in the speech tasks, the effect of the motor movements on learning was stronger in the speech domain. Finally, no correlation between speech and non-speech learning was observed. Overall, our findings support the idea that learning statistical patterns from speech versus non-speech relies on segregated mechanisms, and that the speech motor system contributes to auditory statistical learning in a highly specific manner.

In their day-to-day lives, humans are exposed to an abundance of structured sensory signals, like speech and music. Extracting statistical regularities and patterns in these complex signals is widely referred to as statistical learning (SL) and plays a key role in human learning from the earliest developmental stages (Frost et al., 2019; Krogh et al., 2013; Saffran & Kirkham, 2017). SL plays an essential role in language acquisition (Misyak & Christiansen, 2012; Saffran et al., 1996; Siegelman, 2020) and other cognitive functions (i.e., attention, e.g., Forest et al., 2022; visual search, e.g., Baker et al., 2004; contextual cueing, e.g., Goujon et al., 2015; and motor learning, e.g., Hunt & Aslin, 2001) across different modalities (i.e., auditory, e.g., Lammertink et al., 2019; visual, e.g., Siegelman, Bogaerts, Kronenfeld, et al., 2018; tactile, e.g., Conway & Christiansen, 2005).

Despite extensive evidence of the human ability to discover a wide variety of patterns and regularities in the environment, it is not completely understood how SL is implemented in different modalities (i.e., auditory, visual, or tactile) and domains (i.e., linguistic, or non-linguistic; Conway, 2020; Bogaerts et al., 2022). Some findings support the view that a variety of modality- and domain-specific mechanisms contribute to SL. For instance, auditory statistical learning is easier at a fast than a slow stimulus presentation rate while visual SL is easier at a slow than a fast stimulus presentation rate (Emberson et al., 2011). SL also follows distinct development trajectories across the visual, auditory and motor domain (Arciuli & Simpson, 2011; Janacsek et al., 2012; Raviv & Arnon, 2018), and adults learn better in the auditory modality than in the visual modality despite the similarity of the underlying patterns (Conway & Christiansen, 2005). Recently, Lukics and Lukács (2022) showed that the modality (auditory versus visual) and domain (linguistic versus non-linguistic) of the input stimuli affect SL in an artificial grammar learning task, such that performance is stronger with auditory speech stimuli than with other stimuli (of other modalities and domains) when presented in a similar serial format. Moreover, there exists no or only weak correlations between performance on distinct SL tasks, both within a modality (e.g., auditory SL tasks using different syllables, Erickson et al., 2016) and across modalities (e.g., visual, and auditory SL; Siegelman & Frost, 2015; but see also Siegelman et al., 2018).

In 2015, Frost and colleagues proposed a theoretical framework to explain how SL can operate across different domains and modalities while also demonstrating specificity. More specifically, they argued that SL involves domain-general computational principles that are implemented by distinct neural networks in the brain, depending on the properties of the input signal that is processed (i.e., visual, auditory, or somatosensory). This means that while the computations extracting the regularities might be shared, the neural substrates that implement them are not. Differences in SL across modalities can then be ascribed to the modality-specific encoding constraints (e.g., auditory cortex is sensitive to temporal information but less so for spatial information). This view of a domain-general processing mechanism in concert with modality-specific constraints is echoed by a more recent review of Conway (2020). While acknowledging that the effects of input domain and modality on SL remain an open question for the field, it is likely that a combination of domain-general and domain-specific neural mechanisms operate in interaction, for instance by assuming connections between modality-specific brain regions and the prefrontal cortex (Conway, 2020).

Recent studies have provided compelling evidence for the involvement of auditory-motor mechanisms in auditory statistical language learning (Assaneo et al., 2019; Orpella et al., 2022). Auditory-motor synchronization refers to the ability to coordinate a series of motor gestures (i.e., body movements) with a rhythmic auditory stimulus in the surrounding environment. The ability is regarded as an inherent human trait (Provasi et al., 2014) and subserves various human activities, such as dancing or playing an instrument, as well as the development of intricate cognitive abilities (e.g., reading; Carr et al., 2014; Miura et al., 2015; Zatorre et al., 2007). Notably, Assaneo et al. (2019) demonstrated that individuals who spontaneously synchronize their speech motor movements (repeatedly whispering the sound ‘tah’) with the rhythm of a stream of speech sounds (random syllables) exhibit improved learning outcomes on a distinct statistical language learning task. Additionally, a separate cohort of high-synchronizers also showed stronger entrainment of frontal motor brain areas to speech sound sequences than low-synchronizers. These results were extended by Orpella et al. (2022), who showed that high synchronizers’ learning outcomes were impaired by concurrent whispering during exposure to a stream of speech sounds. While these recent studies provide strong evidence for motor contributions to auditory statistical language learning, they raise questions of specificity. Does the speech motor system play a domain-general role in auditory SL or does the speech motor system play a specific role in acquiring patterns and regularities from sequences of speech sounds specifically? Intriguingly, SL of patterns in tone sequences has also been shown to engage frontal motor regions in the left hemisphere (Moser et al., 2021; Farthouat et al., 2017). This has led to a proposal that the left-lateralized motor system could contribute to auditory SL in a domain-general manner. For example, via the phonological working memory loop (Baddeley et al., 1998), or by predictive mechanisms that modulate auditory processing (Park et al., 2020). On the other hand, there is evidence that the speech motor system affects auditory processing of speech sounds in a highly specific manner (Möttönen et al., 2013; Smalle et al., 2015): (1) disruption of the speech, but not hand, motor regions in the left motor cortex modulates auditory processing of speech sounds, and (2) disruption of the speech motor regions has no effect on auditory processing of piano tones. Although this pattern suggests that auditory-motor processes are domain-specific, it remains to be tested directly whether the motor contributions to auditory SL are also domain-specific, or whether the speech motor system supports computations underlying auditory SL in a domain-general manner. This is an important question to address because auditory-motor synchronization is considered to be a potential useful tool in the clinic. For instance, it has been shown to facilitate speech in non-verbal children with autism (Wan et al., 2011) or can be used to train motor skills (Bood et al., 2013). Hence, testing the specificity of motor contributions to auditory SL may help us understand the precise characteristics and fine-tune implications for the practice (e.g., auditory-motor training in light of language development).

In the present study, we aim to characterize specificity of motor contributions to auditory SL in three experiments. In Experiment 1, we tested specificity of motor processes supporting learning of statistical regularities in speech sequences. Participants performed either a speech motor task (i.e., whispering a syllable) or a non-speech motor task (i.e., clapping hands) during exposure to a structured stream of speech sounds. SL was tested using a two-alternative forced choice recognition task that contrasted patterns from the exposure sequence to novel patterns. In Experiment 2, we focused on auditory specificity. We tested whether a speech motor task (i.e., whispering) affects learning patterns in speech sequences (partial replication of Experiment 1) as well as in non-speech sequences (i.e., tone structures). In Experiment 3, we examined whether learning statistical patterns in speech and non-speech sequences is correlated. If auditory SL is supported by domain-specific auditory-motor processes, then whispering, but not clapping, should impair learning of regularities between speech sounds in Experiment 1, and whispering should have no effect on learning statistical patterns in non-speech sounds in Experiment 2. Moreover, auditory-motor synchronization abilities are expected to differ across the speech and non-speech domain. Additionally, if the processes underlying SL are domain-specific, we expect no correlation in Experiment 3. Overall, this pattern of findings would support the idea that the speech motor system contributes to auditory SL in a domain-specific manner and that learning regularities in speech and non-speech sequences relies on segregated mechanisms.

## Materials and Methods

### Participants

Native-English speaking participants between 18 and 45 years were recruited via the online participant platform Prolific. An a-priori power analysis using the smallest independent effect-size in Smalle et al. (2022) (i.e., d = .6), informed us that we would need 36 participants per between-subject condition. A total of 168 participants completed *Experiment 1*, of which 89 participants in the whispering condition (i.e., performing a speech-motor task) and 97 participants in the clapping condition (i.e., performing a non-speech motor task). *Experiment 2* was completed by 100 participants, of which 41 participants in the speech condition (i.e., listening to speech) and 59 participants in the tone condition (i.e., listening to non-speech). Finally, *Experiment 3* was completed by 101 participants. To ensure high quality of the data included in the analyses, multiple exclusion criteria were used. First, participants had to pass the headphone screening test by Woods et al. (2017) implemented at the start of the experiment. The test is specifically designed for online experiments to identify people who are not wearing headphones (see further details in Tasks). It is important that participants wear headphones to diminish their own auditory feedback of whispering or clapping when listening to the structured sequences (Assaneo et al., 2019; Orpella et al., 2022). The headphone screening test was implemented as offline screening tool for post-hoc exclusion in Experiment 1 and 3. In Experiment 2, it was implemented online to directly exclude participants from further participation. Second, for Experiment 1 and 2, audio recordings of whispering and clapping were checked to ensure that participants performed the motor tasks as instructed. Similar to Assaneo et al., (2019), participants were excluded if (1) they were whispering out loud (or, likewise, clapping too loudly) (2) they stopped whispering (or clapping) for longer than 3 seconds, (3) the recording was contaminated with the auditory stimulus and/or (4) there was persistent background noise. As a result, in *Experiment* 1, we report the data of 68 participants who were randomly assigned to a whispering condition (n = 34, age = 28.2 *_M_* ± 5.9 *_SD_*, 16 females) or a clapping condition (n = 34, age = 27.8 *_M_* ± 7.6 *_SD_*, 18 females). In *Experiment* 2, we report the data of 79 participants who were randomly assigned to a speech condition (n = 33, age = 29.7 *_M_* ± 6.5 *_SD_*, 18 females and 1 genderqueer) or a tone condition (n = 46, age = 31.8 *_M_* ± 7.0 *_SD_*, 21 females). Finally, in *Experiment* 3, we report the data of 80 participants who listened to structured speech and tones (age = 30.7 *_M_* ± 7.5 *_SD_*, 37 females and 3 genderqueers) without motor task. None of the participants reported a history of language (learning) impairments, psychiatric and/or neurological problems. For Experiment 3, 36 participants reported having an autism spectrum disorder.^1^ All participants signed an informed consent form at the start of the experiment and received financial compensation at the end of participation (8£/hour). The experimental procedure received approval from the Research Ethics Committee of the Faculty of Psychology and Educational Sciences at Ghent University (reference: 2021/4 Eleonore Smalle).

### Experimental design

**Figure 1:**
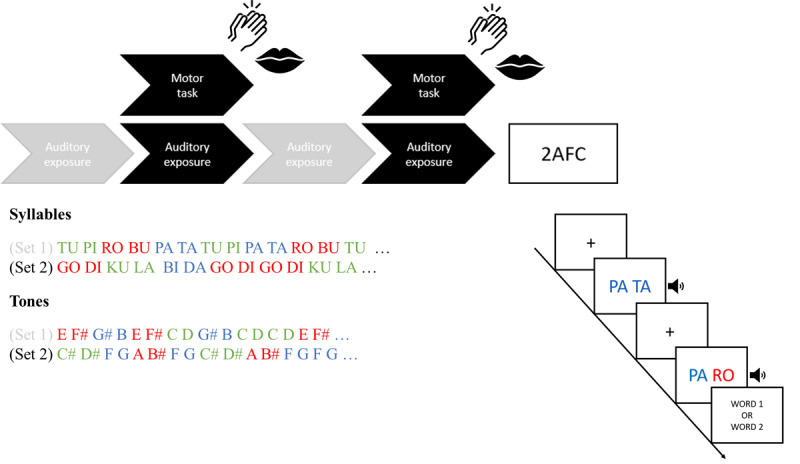
Experimental Design. Experimental protocol. In all three experiments, participants listened to clips of two different structured auditory streams in alternating order. Statistical learning of the hidden structures was tested afterwards via a 2AFC recognition test. In Experiment 1, participants listened to structured syllable streams (syllables set 1 and 2). They were instructed to continuously whisper the speech sound ‘tah’ (speech motor task) or clap hands (non-speech motor task) during the clips of one stream (black). During the clips of the other stream (grey), they were not instructed to perform an additional motor task (only listen). In Experiment 2, participants listened to structured speech (syllables set 1 and 2) or structured non-speech (tones set 1 and 2). They concurrently performed the speech motor task (whispering ‘tah’) during the clips of one stream (black) and only listened to the clips of the other stream (grey). In Experiment 3, participants listened to one structured speech stream (tones set 1 or 2) and one structured syllable stream (syllables set 1 or set 2). No motor task was performed in Experiment 3. Following the exposure phase, participants across all three experiments completed a 2AFC recognition task to measure SL of the sound structures.

All data were collected online via the Gorilla experiment builder (Anwyl-Irvine et al., 2020). The three experiments started with a headphone screening test, after which the main SL task, depicted in Figure 1, was presented. During the main task, participants were exposed to two auditory streams that each contained a continuous repetition of three novel sound structures. The streams were presented via short 1-minute clips in an interleaved design, until each duplet was repeated for 90 times in total (see further in Tasks). In Experiment 1, participants listened to structured syllable streams (speech exposure). They were instructed to perform a concurrent motor task during the clips of one stream and passively listen (i.e., without performing an additional motor task) during the clips of the other stream. More specifically, participants silently whispered the syllable ‘tah’ (speech motor task) or clapped their hands (non-speech motor task) continuously for one minute when prompted. In Experiment 2, participants listened to structured syllable streams (speech exposure) or structured tone streams (tone exposure). They were all instructed to silently whisper the syllable ‘tah’ (speech motor task) during the clips of one stream and passively listen to the clips of the other stream. Finally, in Experiment 3, all participants were exposed to a structured syllable stream and a structured tone stream without motor task. In all experiments, the order of the streams (and concurrent motor task) was counterbalanced across participants such that all possible combinations occurred. Following the exposure phase, participants across all three experiments completed a two-alternative forced choice (2AFC) recognition task to measure SL of the sound structures. At the end of the experiment, participants were asked about audio quality and wearing their headphones. All included participants confirmed good audio quality and reported wearing headphones as instructed. The entire procedure took approximately 15 to 25 minutes to complete.

### Tasks

#### Headphone screening test

Three 200 Hz pure tones were played sequentially for 1000 ms at an inter-stimulus interval of 500 ms. The instruction was to indicate the quietest sound. One of the tones was played at a lower intensity (– 6 dB) compared to the other two tones. Of the tones with equal intensity, one was presented 180 degrees out of phase across stereo channels. This misleadingly attenuates the tone for the listener that is not wearing headphones. The task thus helps discriminating between participants wearing headphones and participants who are not (Woods et al., 2017). All participants completed six trials of the hearing test before commencing the main experiment. A criterium of five correct trials was used to pass the screening.

#### Exposure task

Participants were exposed to a total of six novel sound structures, which were presented along two structured streams. In Experiment 1, participants were exposed to two streams of three repeating syllable duplets. The duplets were constructed out of 12 unique syllables with a consonant-vowel structure and matched across streams on English phonotactic probability (p = .9) (i.e., stream set 1: /tu:pɑI/, /roʊbu:/ and /pɑ:tɑ:/; stream set 2: /ku:lɑ:/, /goʊdɪ:/ and /bi:dɑ:/). In Experiment 2, participants were exposed to two streams of three repeating syllable duplets (same as in Experiment 1) or two streams of three repeating tone duplets. The tone duplets were constructed out of 12 pure tones within the same octave (considering the English notation scheme, A at 440 Hz) and matched across streams on frequency interval (i.e., stream set 1: [CD], [EF#] and [G#B]; stream set 2: [C#D#], [FG] and [AB#]). In Experiment 3, participants were exposed to a stream of three repeating syllable duplets and a stream of three repeating tone duplets (i.e., stream set 1 from the previous two experiments). All syllables were generated using an online artificial speech synthesizer of a female British English voice. The tones were generated via Praat (Boersma & Weenink, 2023). The sound files were edited and saved with a sampling rate of 44 100 Hz using Audacity software. In the present study, we chose duplet stimuli rather than triplet stimuli, which have been typically used in previous studies. This was done to keep the online experiments short and feasible. Piloting and results from our main experiments confirm that learning performance for three duplet structures in two conditions is similar to learning performance for four triplet structures (~60%–70%; e.g., Batterink & Paller, 2017; Smalle et al., 2022; Orpella et al., 2022).

During exposure, the duplets within each stream were repeated for a total of 90 times in a pseudorandom order. The constraint was that the same duplet could not repeat itself more than twice such that the transitional probability between sounds within duplets was 100% while it was 33% between duplets (e.g. /tu:/ was always followed by /pɑI/ while /pɑI/ could be equally followed by /roʊ/, /pɑ/ or /tʏ/). In Experiment 1, the syllables were continuously presented at a rate of 4.5 Hz (i.e., each sound had a duration of 222 ms) analogous to the average speech rate across languages (Ding et al., 2017; Varnet et al., 2017). The streams were split in shorter clips and presented alternatingly during the exposure phase and all duplets were tested at the end of exposure (see Figure 1). Interleaved exposure of two streams was chosen to avoid changes in awareness of the underlying structure of the streams and tasks.

In Experiment 2, the syllables and tones were continuously presented at a rate of 1.8 Hz (i.e., each sound had a duration of 550 ms). We slowed down the rate because pilot testing revealed no tone learning at a higher presentation rate. Nevertheless, the interesting prediction can be made that the motor task affects learning also when stimuli are presented at a slower rate, showing robustness of the effect (partial replication of Experiment 1). The streams were again presented alternatingly via four clips of approximately 1 minute and 15 seconds (see Figure 1). Finally, in Experiment 3, the syllables and tones were presented at their optimal rate (i.e., 4.5 Hz for the syllables and 1.8 Hz for the tones). The syllable stream was presented via two 1-minute clips. The tone stream was presented via two clips of approximately 2 minutes and 30 seconds. These clips were again presented in alternating format (Figure 1).

The motor task was practiced via an example first. During the practice, participants listened to a 1-minute recording of either continuous whispering (speech motor task) or continuous clapping (non-speech motor task). In these examples, the whispers and claps were presented at the same rate as the stimuli in the auditory streams during exposure. Participants were then asked to practice by recording their own clip. At the start of the exposure, the participants were told to listen carefully to the sounds and to perform the instructed motor task when prompted on the screen. The textual prompts “listen only”, “whisper tah” or “clap your hands” were presented simultaneously with the audio-files during the exposure.

#### Recognition task

On each trial, a fixation cross appeared for 500 ms after which participants were presented with two audio files with an inter-stimulus interval of 1500 ms. One audio file contained the duplet from the exposure and one contained a foil duplet. The foils were created from the same stimuli used in the exposure stream but the stimuli never succeeded one another, not even across duplet boundaries (e.g. /tu:tɑ:/, /pɑ:roʊ/, etc). As shown by Siegelman et al. (2017), contrasting words with ‘non-word’ foils better discriminates between low and high statistical learners than contrasting words with partword foils. To further minimalize stimulus-driven effects, each of the target duplets was paired exhaustively with each of the foils by using a Latin squared design (e.g., Siegelman & Frost, 2015; Batterink & Paller, 2017; Smalle et al., 2022). The position of the target in the trial (first or second presentation) was also counterbalanced. As a result, there were 18 recognition trials per stream (3 structures × 3 foils × 2 order presentations), and thus 36 recognition trials in total per participant. The trials were presented randomly to each participant. The instruction was to indicate (1) which of the two structures sounded most familiar, and (2) their confidence for the chosen structure (1: “*I remember the structure from the sound streams*”, 2: “*I have the feeling that the structure sounds familiar but I don’t remember it*”, 3: “*I guessed and have no idea which structure appeared in the sound stream*”). We did not have any clear hypotheses about the confidence ratings but present them in supplementary file. There was no time limit and the next trial started after providing a response.

### Statistical analysis

The main analyses were performed on data from the 2AFC recognition task. One-sample t-tests (one-tailed) were conducted to check whether participants perform above the 50% chance level, indicating SL. Two-tailed independent-t tests were performed across groups. For experiment 1 and 2, logistic mixed effects models were specified using the lme4 package in R (Bates et al., 2014). Recognition trial accuracy (correct/false) was entered as dependent variable, Condition (passive listening versus motor task), Group (i.e., Experiment 1: whispering versus clapping; Experiment 2: speech versus tones) and Stream (stream 1 or stream 2), and their interaction were entered as fixed factors. For model selection, we followed the guidelines used in Singmann & Kellen (2019). We always tried to include the maximal random effects structure in the models justified by the design. In the case of convergence issues, such as singular fits, we refitted the maximal model by first removing correlations among random slopes. Following the correlations, we removed the highest-order random slopes with the least estimated variance (Baayen et al., 2008; Jaeger, 2008). We additionally compared the maximal models to simpler models by using Akaike Information Criterion (AIC). If a simpler model showed better fit to the data, then the simpler model was chosen. For Experiment 1, the final converging model was Accuracy ~ Stream * Condition * Group + (1|Subject). For Experiment 2, the final converging model was Accuracy ~ Condition * Group + (1 |Item) + (1 | Subject). Effect coding was used for all factors. All p-values were derived using the Kenward-Roger approximation for degrees of freedom with the ANOVA function in the afex package (Singmann et al., 2015). Significant effects were followed up with t-tests from the R package stats. For the t-tests, Cohen’s deffect sizes are reported. For Experiment 3, Pearson correlation analyses were conducted between participants’ learning performance for speech sounds and tones. All data and analysis scripts are available on the Open Science Framework (https://doi.org/10.17605/OSF.IO/6BKH3).

We additionally assessed participants’ spontaneous auditory-synchronization abilities during the exposure phases of Experiment 1 and 2, by calculating phase-locking values (PLVs) between the participants’ produced motor action (Experiment 1: whispering or clapping; Experiment 2: whispering) and the perceived stimuli (syllables in Experiment 1; syllables or tones in Experiment 2) from the recordings that we obtained. While spontaneous speech motor synchronization is typically measured in a separate test with random syllables (e.g., SSS-test in Assaneo et al., 2019), recent work showed that synchronization abilities do not differ when assessed during a learning phase with structured syllables (Orpella et al., 2022). Two-sample t-tests are performed in each experiment to compare the obtained PLVs across task conditions (i.e., Experiment 1: whispering to speech vs. clapping to speech; Experiment 2: whispering to speech vs. whispering to tones). We also performed exploratory K-means clustering analyses (k = 2) on the PLV data, similar to the procedure in Assaneo et al. (2019), to dissociate high and low synchronizers. The histograms and obtained bimodal distributions of Experiment 1 and 2 are provided in a supplementary file (Supplementary Results S1). To test whether the effect of whispering affected SL from speech streams specifically in the group of high synchronizers, similar to the original findings of Orpella et al. (2022), we pooled the whispering data from Experiment 1 and 2 (Supplementary Results S2).

## Results

### Whispering but not clapping impairs auditory statistical learning from speech sequences (Experiment 1)

**Figure 2:**
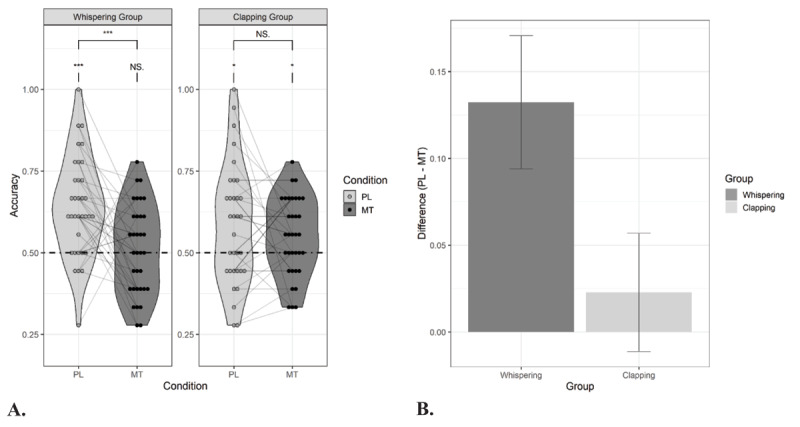
Performance in the forced-choice recognition task in Experiment 1. **Panel A:** Proportion correct responses for speech duplets that were presented under a passive listening (PL) and motor task (MT) condition during exposure. Thirty-four participants performed a concurrent speech motor task (whispering) and thirty-four participants performed a concurrent non-speech motor task (clapping). The dashed line indicates chance level performance. Asterisks denote significance for one-sample t-tests and paired t-tests: *p < .05, **p < .01, ***p < .001, NS. = non-significant. **Panel B:** The effect of the motor task on statistical learning (i.e., accuracy for passive listening condition – accuracy for motor task condition) for both the whispering group and clapping group.

The results of the learning performance in Experiment 1 are presented in Figure 2. All participants showed learning in the condition without motor task, indicating baseline SL (i.e., whispering group: M = 0.64, SE = 0.03, *t*(33) = 5.44, *p* < .001, *d* = 0.93; clapping group: M = 0.57, SE = 0.03, *t*(33) = 2.17, *p* = .02, *d* = 0.37; Group comparison: *t*(62.61) = –1.66, *p* = .10, *d* = –0.40). The concurrent motor task significantly impaired learning (i.e., main effect of Condition: *β* = 0.16, SE = 0.04, *Z* = 3.93, *X^2^* (1) = 15.45, *p* < 0.001, estimate’s effect size = 0.33). Importantly, the impairment was stronger in the whispering group than in the clapping group (i.e., Group × Condition interaction: *β* = –0.12, SE = 0.04, *Z* = –2.91, *X^2^* (1) = 8.48, *p* = .003). Planned paired t-tests (two-tailed) showed a significant reduction in learning in the whispering group (i.e., listening versus whispering: *t*(33) = 3.45, *p* < .001, *d* = 0.56) but no evidence for that in the clapping group (*t*(33) = 0.67, *p* = .26, *d* = 0.10). Learning was above chance when clapping (M = 0.55, SE = 0.04, *t*(33) = 2.39, *p* = .01, *d* = 0.41) but not when whispering (i.e., M = 0.51, SE = 0.02, *t*(33) = 0.42, *p* = .34, *d* = 0.10).

### Whispering impairs auditory statistical learning from speech but not from tone sequences (Experiment 2)

**Figure 3:**
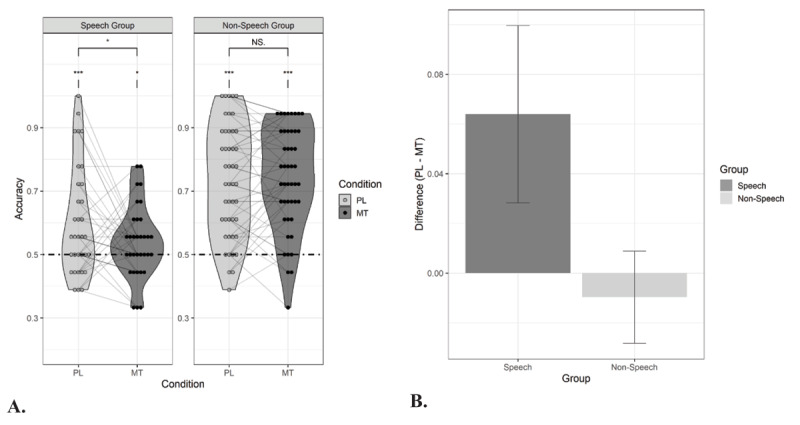
Performance in the forced-choice recognition task in Experiment 2. **Panel A:** Proportion correct responses for sound duplets that were presented under passive listening (PL) and motor task (MT) conditions during exposure. In Experiment 2, the motor task condition was the same for both groups, namely whispering the syllable ‘tah’. Thirty-three participants listened to duplets of syllables (Speech Group) and forty-six participants were exposed to tone duplets (Non-Speech Group). The dashed line indicates chance level performance. **Panel B:** Visualization of the suppressive effect of the motor task on statistical learning (i.e., accuracy for passive listening condition – accuracy for motor task condition, here whispering) for both the Speech group and Non-Speech group. Asterisks denote significance for one-sample t-tests and paired t-tests: *p < .05, **p < .01, ***p < .001, NS. = non-significant.

The results of the learning performance in Experiment 2 are presented in Figure 3 Again, all participants showed baseline learning (i.e., speech group: M = 0.60, SE = 0.03, *t*(32) = 3.47, *p* < .001, *d* = 0.60; tone group: M = 0.73, SE = 0.03, *t*(45) = 8.95, *p* < .001, *d* = 1.32), but recognition of the duplets was higher for the tone group than for the speech group (i.e., *t*(69.08) = 3.21, *p* = .002, *d* = 0.73). Baseline performance in the speech group did not significantly differ from baseline performance in the speech group of Experiment 1, despite the slower presentation rate for the syllables in Experiment 2 (i.e., *t*(63.48) = –0.95, *p* = .35). Logistic mixed effects modelling revealed a main effect of Group (*β* = 0.42, SE = 0.08, *Z* = 5.08, *X^2^* (1) = 25,78, *p* < .001, estimate’s effect size = 0.85) but no main effect of Condition (*β* = 0.07, SE = 0.04, *p* = .12). The interaction between Group and Condition was significant (i.e., *β* = –0.08, SE = 0.04, *Z* = –1.93, *X^2^* (1) = 3.73, *p* = .05). Planned paired t-tests showed a significant reduction in performance as a result of whispering in the speech group (listening vs. whispering: *t*(32) = 1.79, *p* = .04, *d* = 0.44) but no evidence for that in the tone group (*t*(45) = –0.52, *p* = .70, *d* = –0.06). Learning remained above chance in both groups (i.e., speech group: M = 0.54, SE = 0.02, *t*(32) = 2.04, *p* = .02, *d* = 0.35; tone group: M = 0.74, SE = 0.02, *t*(45) = 10.14, *p* < .001, *d* = 1.50).

### Spontaneous auditor-motor synchronization is higher in the non-speech than in the speech domain (Experiment 1 and 2)

Additional phase-locking value analyses show that synchrony to speech was higher for the clapping group (mean PLV = .63, SD = .26) than for the whispering group (mean PLV = .41, SD = .19, Welch *t*_60.2_ = 4.09, *p* < .001) in Experiment 1, and higher for whispering to tones (mean PLV = .58, SD = .19) than for whispering to speech (mean PLV = .51, SD = .15; Welch *t*_76.7_= 4.09, *p* = .035) in Experiment 2. Supplementary analyses (S1) show the results from exploratory K-means clustering analyses (k = 2) on the obtained PLV values and the effect of whispering on high versus low synchronizers when pooling the data across Experiment 1 and 2.

### No correlation between statistical learning from speech and non-speech sound sequences (Experiment 3)

For both syllable and tone sequences, learning performance was above chance as showed by one-sample t-tests (one-tailed) (*M*_syllable_ = 0.56, *SE* = 0.02, *t*(79) = 3.18, *p* = 0.001, *d* = 0.36; *M*_tone_ = 0.76, *SE* = 0.02, *t*(79) = 14.91, *p* < 0.001, *d* = 1.67). Accuracy in the 2AFC task was however significantly lower when learning syllable duplets compared to learning tone duplets, as shown by the paired sample t-test (two-tailed) (*M*_difference_ = –0.20, *SE* = 0.02, *t*(79) = –8.30, *p* < 0.001, *d* = 1.27). Additionally, SL performance on syllable and tone sequences was not correlated (r(78) = 0.06, *p* = 0.58; self-reported autism: r(34) = .07, *p* = .67; no autism: *r*(42) = .06; *p* = .71). This was supported by an additional Bayesian analysis providing moderate evidence for the absence of a relationship (BF_01_ = 3.38).

## Discussion

In the present, study we investigated the domain-specificity of motor contributions to auditory SL. We manipulated the involvement of the motor system in auditory processing by concurrent motor tasks, engaging either the vocal tract or hands. We present evidence that a concurrent speech motor task impairs SL from speech streams, in agreement with previous studies (Lopez-Barroso et al., 2011; Orpella et al., 2022). Importantly, we also present new evidence that this motor contribution to auditory statistical language learning is specific to the speech-domain. An additional motor task that involved speech (i.e., whispering) negatively impacted SL of speech sounds more than an additional motor task that did not involve speech (clapping, i.e., Group × Condition interaction in Experiment 1). Moreover, an additional speech motor task negatively impacted SL of speech sounds more than SL of non-speech sounds (i.e., Group × Condition interaction in experiment 2). Additional phase-locking value analyses showed that spontaneous auditory-motor synchronization during the exposure is stronger for participants in the non-speech tasks (clapping to speech or whispering to tones) than for participants in the speech tasks (whispering to speech). In Experiment 3, an individual’s ability to learn statistical regularities from speech streams during passive listening was not correlated with their ability to learn statistical regularities from tone streams, which was further confirmed by a Bayesian analysis. It should be considered that performance was higher in the non-speech task than in the speech task, and thus different levels of task complexity could have engaged different mechanisms and alternatively explain the lack of correlation. Together, our findings suggest that speech-related auditory-motor mechanisms are critical for statistical language learning, but that the involvement depends on acoustic characteristics of the auditory stream and/or the effector, i.e., the motor contributions are domain-specific.

Auditory-motor synchronization is considered to be an inherent trait of humans, assuming that any individual can synchronize at some degree to any sound (e.g., Mares et al., 2023). The recent work by Assaneo et al., (2019) and Orpella et al. (2022) suggest a division in the general population in speech domain, with only one sub-group of individuals demonstrating high synchrony of the produced syllables to the perceived syllabic rate, which relates to statistical language learning abilities. The pattern of our results indicates a specific and restricted role of the speech motor system in auditory SL, emphasizing its domain-specific nature. While the speech motor system is indeed involved in auditory SL, similar to what is observed with the effect of articulatory suppression in Orpella et al. (2022), our findings propose a stronger involvement of the system in learning speech structures compared to non-speech structures. Additionally, we observe that although participants exhibit high capability in synchronizing hand movements with the heard speech rate, the hand motor system is not as involved in learning speech structures as the speech motor system is. These findings suggest a more restricted role of auditory-motor mechanisms in statistical language learning than previously thought. This nuanced understanding could have significant implications for the development of auditory-motor entrainment therapies in clinical settings.

The study by Orpella et al (2022) showed that whispering affected SL from speech streams specifically in the group of high synchronizers, but not in low synchronizers. A supplementary analysis in the current study showed that whispering affects learning in both high and low synchronizers. There was however a non-significant trend towards a stronger effect in high than low synchronizers. Methodological differences between studies could potentially explain the differences in results. For example, Orpella et al (2022) used a separate test to classify participants to high and low synchronizers in a laboratory setting, which was not included in the current online study. The findings by Orpella et al. (2022) also raise the question whether higher synchronization in speech conditions than non-speech conditions could potentially explain the speech-specific motor contributions in the current study. Our phase-locking value analyses do not support this explanation. In Experiment 1, spontaneous auditory-motor synchronization was higher during clapping than whispering when listening to speech streams. In Experiment 2, synchronization was higher when listening to non-speech than speech streams when whispering. Interestingly, these results suggest that both the nature of the motor task and auditory stimuli affected an individual’s spontaneous ability to synchronize motor movements with auditory streams: auditory-motor synchronization was stronger in the non-speech than speech domain. In contrast, the effect of motor movements on auditory SL was strongest in the speech domain. It should be noted that the current study used a between-subjects design and therefore the differences in synchronization could potentially be partly explained by individual differences in synchronization ability between non-speech and speech conditions. Recent work by Mares et al. (2023) suggests that individual synchronization ability remains quite stable across different effectors (vocal tract vs. hands) and stimulus types (syllables vs. tones), but that there are some differences between high and low synchronizers. Future studies should use a within-subjects design to investigate the domain-specificity of spontaneous auditory-motor synchronization and SL.

In the present study, we presented speech sounds at a frequency of 4.5 Hz, which is a natural speech rate across languages (Ding et al., 2017; Poeppel & Assaneo, 2020) and has been shown to be optimal for auditory-motor coupling in the speech domain (Assaneo & Poeppel, 2018). In Experiment 2, however, we had to lower the rate to 1.8 Hz to match the presentation rate of speech and tones. Whispering reduced SL from speech streams in both experiments, indicating that motor contributions to learning of statistical regularities are robust across speech rates. In general, participants showed weaker SL performance for syllable compared to tone streams during passive listening (Experiments 2 and 3). One reason for this difference could be the prior expectations of participants regarding the co-occurrence of syllables (Siegelman et al., 2018). Performance on an auditory SL task involving verbal material does not only reflect how well participants compute statistical regularities but also how they update existing expectations regarding syllable co-occurrences (note that care was taken to match the words and non-words in this experiment on average phonotactic probability). As tones do not invoke strong prior expectations regarding co-occurrences, the lack of need for updating could be reflected in the learning outcomes.

In contrast to previous findings, learning was completely absent (i.e., at chance level) during the whispering condition in Experiment 1. Exploratory Bayesian analyses confirmed moderate evidence for the absence of learning (i.e., one-sample t-test: BF_01_ = 3.83). In Orpella et al. (2022), learning was diminished during whispering but still above chance. We have no clear explanation for why learning was completely abolished because of concurrent whispering in Experiment 1. Note that in Experiment 2, learning performance during whispering was above chance in the speech group (BF_10_ = 2.24, i.e. weak evidence for learning) and the tone group (BF_10_ = 4.78 × 1010, i.e., strong evidence for learning).

Our primary measure of learning was accuracy in the 2AFC task in which participants had to select which syllable/tone duplet was presented during exposure to auditory streams. In addition to previous work, we also measured participants’ confidence in their selection without prior hypotheses. Confidence ratings are often included in statistical learning experiments to learn more about the participants’ knowledge of the acquired patterns. In recent work, Batterink and colleagues showed that even without intention to learn, adults acquire mainly explicit knowledge of the novel word forms during statistical learning (Batterink et al., 2015a; 2015b; 2017, 2019). This can be derived from a correlation between confidence and accuracy on the recognition task, with the absence of a correlation indicating the absence of explicit knowledge. In the current study (Supplementary Results S3), we also observed that high accuracy is associated with high confidence when the duplets are presented during passive listening, indicating explicit knowledge of the structure of the speech streams. Interestingly, this association appears to be absent in the same group of participants when duplets were presented during the concurrent speech motor task. Although speculative, this zero correlation suggests that the concurrent speech motor task abolished participants’ ability to acquire explicit linguistic knowledge. The speech motor task indeed reduced mean confidence in Experiment 1, but this effect was not replicated in Experiment 2. Further research is needed to investigate the role of the speech motor system in acquisition of explicit knowledge from speech streams.

### Further speculation and interpretation of the main findings

The speech motor system is tightly linked to the auditory system, and it contributes to processing of speech sounds, modulating speech perception (for reviews, see Liebenthal & Möttönen, 2018; Skipper et al., 2017). Evidence from combined brain stimulation and electroencephalography studies shows that speech motor and auditory systems interact from the early stages of speech sound processing and that these early auditory-motor interactions are speech-specific (Möttönen et al., 2013). Moreover, evidence from neuroimaging studies shows that the speech motor system is engaged in encoding distinctive features of speech sounds (e.g., Du & Zatorre, 2017; Evans & Davis, 2015; Pulvermüller et al., 2006). One possibility is that in the present study the concurrent speech motor task specifically disrupted early-level auditory-motor processing of speech sounds, which impaired participants’ ability to learn statistical regularities in speech streams. During processing of structured auditory streams, neural oscillations are entrained to their repeating statistical structures as well as their elements, either syllables or tones (Batterink et al., 2015b, 2019; Batterink & Paller, 2017; Bogaerts et al., 2020; Doelling & Assaneo, 2021; Moser et al., 2021). The prevalent hypothesis is that neural oscillations reflect phases of high and low excitability of the neurons involved (Giraud & Poeppel, 2012; Lakatos et al., 2005; Ten Oever & Martin, 2021). Stimulus processing can be enhanced by aligning these phases of high excitability with the edges of the stimuli comprising the stream (e.g., syllables, tones; Lakatos et al., 2005). According to an increasingly popular view, the motor regions form a prediction of the timing of an upcoming stimulus which is communicated to the auditory cortex, modulating the entrainment of the neural oscillations to periodic and quasi-periodic auditory signals (Patel & Iversen, 2014; Rimmele et al., 2018). The speech motor system has also been proposed to synchronize with the auditory system while processing syllable streams. Moreover, this coupling between brain areas is restricted to the lower stimulus rates and is especially enhanced at a stimulus presentation of 4.5 Hz (Assaneo & Poeppel, 2018; Poeppel & Assaneo, 2020). Evidence shows that high synchronizers show stronger brain-to-stimulus entrainment in the left inferior frontal cortex compared to low synchronizers. Additionally, high synchronizers outperform low synchronizers on a subsequent SL task (Assaneo et al., 2019). It is possible that in the present study the speech motor task disrupted temporal processing of speech sound sequences either by weakening generation of temporal predictions or by suppressing the neural oscillator in the speech motor cortex. Future studies are needed to investigate how speech motor system contributes to neural entrainment to structured sound streams and whether these modulations are associated with the ability to extract statistical regularities from speech streams.

Auditory SL relies on two important mechanisms, namely the encoding of the individual elements and the extraction of the dependencies between elements (Thiessen et al., 2013). It has been argued that sensitivity to transitional probabilities between elements in a continuous signal reflects an underlying chunking mechanism, by which adjacent elements are grouped into increasingly higher-ordered representations that receive activation every time they are encountered. Within this view, representations of groupings across (word) boundaries will show less activation because they are re-encountered less frequently during exposure and suffer in competition with representations of groupings within (word) boundaries (Erickson & Thiessen, 2015; Perruchet & Vinter, 1998). This means that whereas learners may appear sensitive to transitional probabilities, they are storing chunks of the input stream, which – owing to interference in memory – are biased toward those statistically coherent chunks that are frequently encountered during exposure (Erickson & Thiessen, 2015). The formation by chunks seems to depend, to some degree, on working memory capacity (see for instance, Janacsek & Nemeth, 2013; Smalle et al. 2016) such that individual elements need to be maintained in working memory for a reasonable amount of time before they can be clustered together. This process relies on a subvocal rehearsal system according to the modality of the auditory information that needs to be retained (e.g., phonological loop or visuospatial sketchpad; Baddeley et al., 1998). The role of subvocal rehearsal system, such as the phonological loop, in retaining auditory information is also supported by studies showing involvement of the speech production system (e.g., left inferior frontal gyrus) in tasks that require phonological storage (Baldo & Cronkers, 2006; Deschamps et al., 2020; Marvel & Desmond, 2012; Shen et al., 2015). Based on this literature, one could speculate that the speech motor system specifically supports chunking of syllables and storing of frequently occurring chunks in memory. This needs to be investigated in future experiments.

## Conclusion

To summarize, the present findings provide support for the view that auditory-motor processing of speech sounds contributes to human ability to learn statistical regularities from speech sound sequences. Although SL can be characterized as a set of domain- and modality-general computational principles, it relies on segregated domain-specific mechanisms even within a single modality.

## Data Accessibility Statement

The data of all three experiments together with the analysis scripts are available on the Open Science Framework (https://doi.org/10.17605/OSF.IO/6BKH3).

## Additional File

The additional file for this article can be found as follows:

10.5334/joc.351.s1 Supplemental Materials. Analysis of participants’ phase-locking values in relation to their learning performance and an analysis of participants’ confidence ratings following speech-specific motor suppression.

## Footnotes

The experiment was part of another study aimed at testing SL differences in self-reported autism. However, since no significant learning differences were found between typical adults (n= 44; speech sounds: M = 0.56, SE = 0.02; tones: M = 0.74, SE = 0.024) and adults with self-reported autism (n = 36, speech sounds: M = 0.55, SE = 0.032; tones: M = 0.77, SE = 0.025) data were pooled for the purpose of a correlational analysis. For completeness, we also report the correlation separately for both groups in the result section.
