# Supplementary material for: Specificity of Motor Contributions to Auditory Statistical Learning
Source: J Cogn. 2024 Feb 16;7(1):25. doi: 10.5334/joc.351 (PMC10870951; doi:10.5334/joc.351)

Supplemental Materials

Histograms and bimodal distributions of the PLVs based on a k-means clustering algorithm (see Methods) (S1)

A k-means clustering algorithm was applied using a squared Euclidian distance metric with 2 clusters, similar to Assaneo et al. (2019).

For Experiment 1, this shows that there are 22 low synchronizers (mean PLV: .28; SD = .08) and 12 high synchronizers (mean PLV: 0.63; SD = 0.11) in the whispering group (Figure left), but 12 low synchronizers (mean PLV: .32; SD = .14) and 22 high synchronizers (mean PLV: 0.80; SD = 0.10) in the clapping group (Figure right). For Experiment 2, the bimodal distribution shows 17 low synchronizers (mean PLV: .39; SD = .06) and 16 high synchronizers (mean PLV: 0.64; SD = 0.09) in the speech group (Figure left). There are 28 low synchronizers (mean PLV: .44; SD = .09) and 18 high synchronizers (mean PLV: .79, SD = .10).

Experiment 1 (left: whispering, right: clapping)

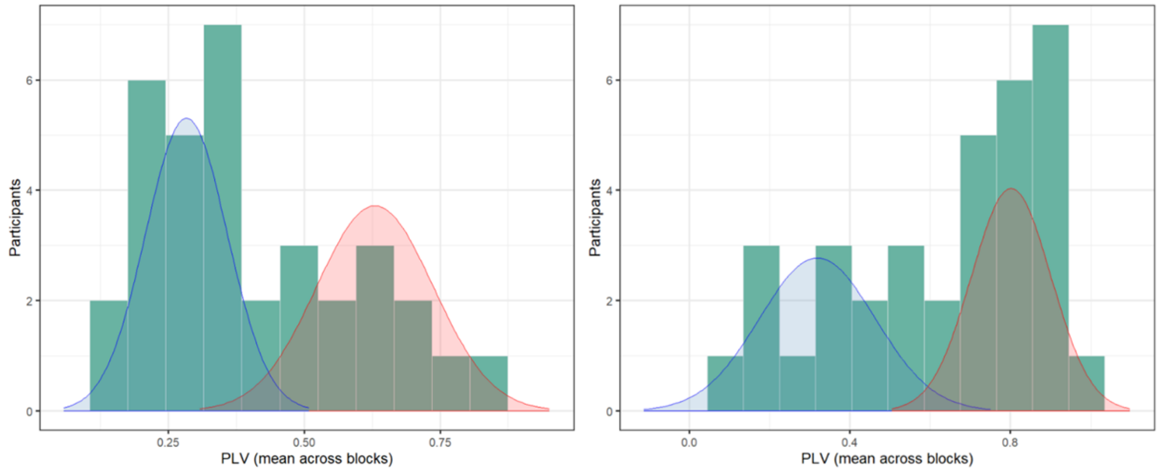

Experiment 2 (left: speech sounds, right: non-speech sounds)

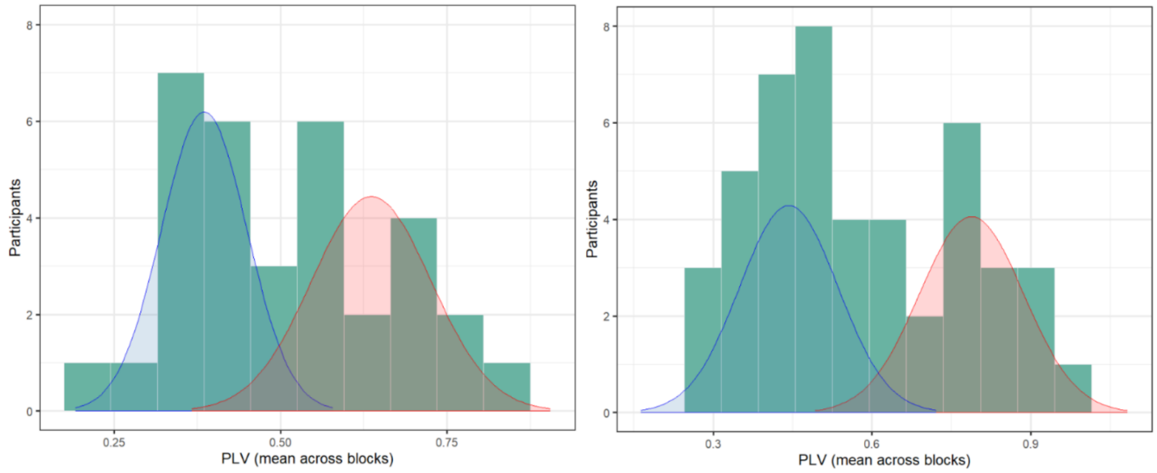

Effect of whispering on auditory statistical learning in high (N = 28) versus low synchronizers (N = 39) (pooled across Experiment 1 and 2) (S2)

The results of the learning performance in the pooled whispering data of Experiment 1 and 2 are presented in the Figure below. All participants showed learning in the condition without speech motor task, indicating baseline SL (i.e., high synchronizers: M = 0.64, SE = 0.03, $t(27) = 4.46$, $p < .001$, $d = 0.84$; low synchronizers: M = 0.61, SE = 0.02, $t(38) = 4.33$, $p < 0.001$, $d = 0.69$; Group comparison: $t(54.77) = -0.89$, $p = .38$, $d = 0.22$). The speech motor task significantly impaired learning (i.e., main effect of Condition: $\beta = 0.22$, SE = 0.04, $Z = 5.17$, X^2 (1) = 26.33, $p < 0.001$, estimate's effect size = 0.68). The impairment was not significantly stronger in the high synchronizers than in the low synchronizers (i.e., Group x Condition interaction: $\beta = 0.03$, SE = 0.04, $Z = 0.80$, X^2 (1) = 0.64, $p = .42$). Planned paired t-tests (two-tailed) showed a significant reduction in learning in the high synchrony group [i.e., listening versus whispering: $t(46.11) = 3.39$, $p = 0.001$, $d = 0.91$] as well as in the low synchrony group [$t(74.68) = 2.28$, $p = .03$, $d = 0.51$]. Learning was above chance when whispering in both groups (i.e., high: M = 0.58, SE = 0.02, $t(55) = 3.76$, $p < 0.001$, $d = 0.50$; Low: M = 0.57, SE = 0.02, $t(77) = 4.14$, $p < 0.001$, $d = 0.47$).

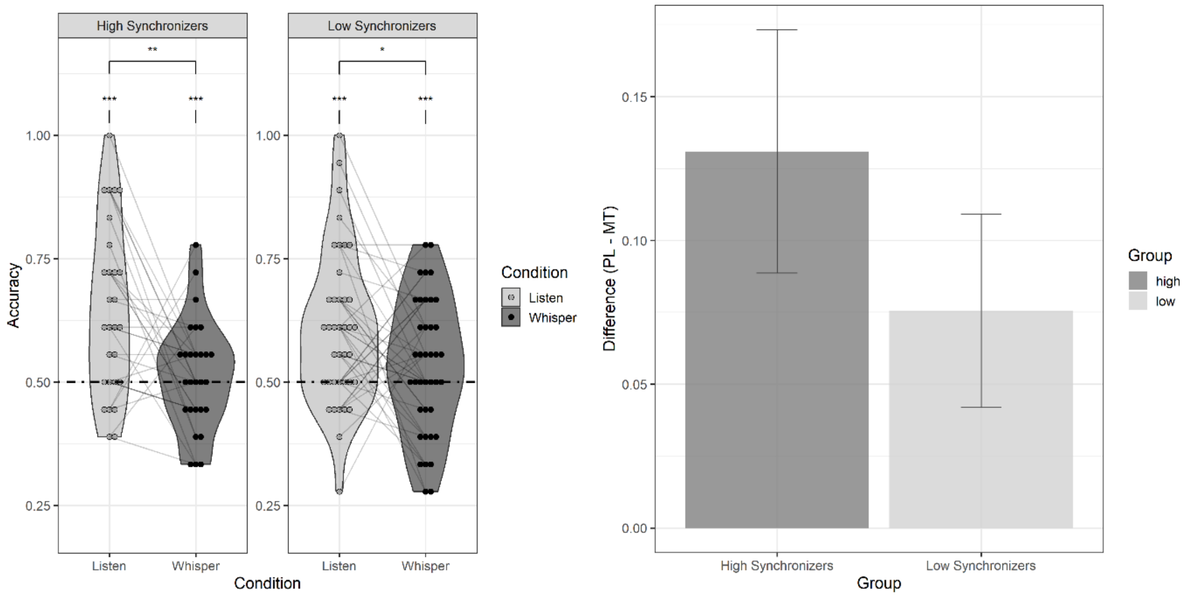

Whispering modulates the relationship between accuracy and confidence (Experiment 1 and 2) (S3)

We exploratory looked at the participants' confidence ratings in the word recognition task. Although the confidence rating was added without a clear hypothesis, one can question whether statistical learning is explicit in nature (e.g., Batterink et al., 2015, 2017; 2022) and whether this is modulated by the motor suppression. For each subject, we averaged confidence scores for correct trials, and performed paired sample t-tests across the passive listening condition and the motor suppression condition. This shows that whispering significantly reduced confidence in memory of the speech structures in Experiment 1 (i.e., M_{PL} = 2.1, SE = 0.07 vs. M_{MT} = 1.8, SE = 0.07, $t(66) = 2.75$, $p < .01$, $d = 0.67$) but not in Experiment 2 (M_{PL} = 2.3, SE = 0.08 vs. M_{MT} = 2.2, SE = 0.06, $t(64) = 1.2$, $p = .23$, $d = 0.3$). Clapping did not affect confidence (i.e., Experiment 1: M_{PL} = 2.2, SE = 0.08 vs. M_{MT} = 2.0, SE = 0.07, $t(66) = 1.91$, $p = .06$, $d = 0.46$). Moreover, whispering did not affect confidence in memorizing tone structures (M_{PL} = 2.1, SE = 0.06 vs. M_{MT} = 2.1, SE = 0.06, $t(90) < 1$, $p = 0.64$, $d = 0.09$). When pooling speech learning data across experiment 1 and 2 (N = 65), additional Pearson correlation analyses indicated a significant correlation between confidence rating and accuracy in the passive listening (PL) condition (r(65) = 0.31, p = 0.01) but not in the motor task condition (MT), i.e. whispering (r(65) = 0.047, p = 0.71), see Figure below.

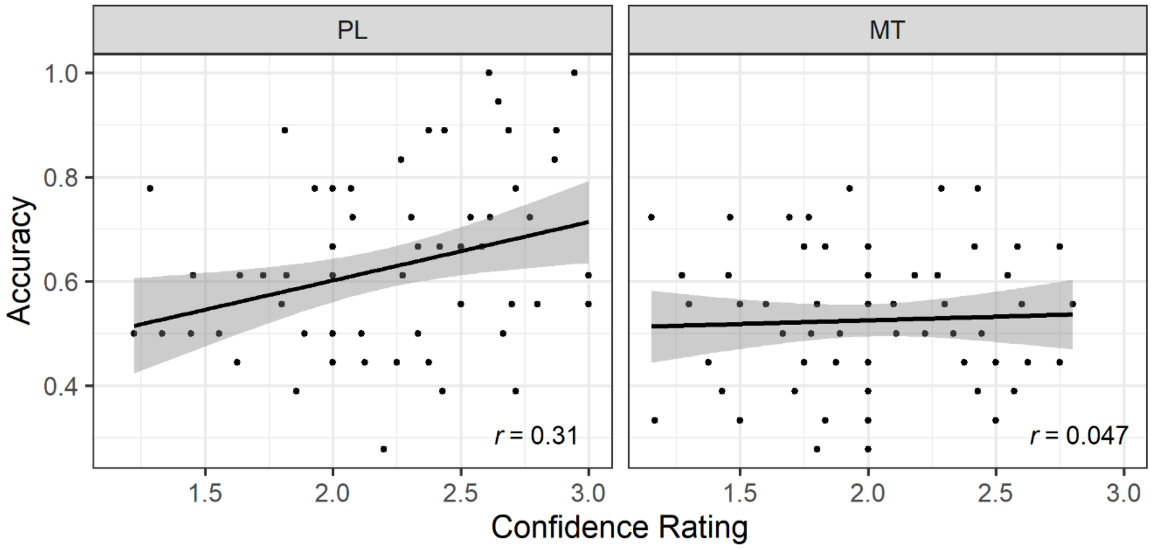

Supplement: Supplemental Materials. — Analysis of participants’ phase-locking values in relation to their learning performance and an analysis of participants’ confidence ratings following speech-specific motor suppression. [file joc-7-1-351-s1.pdf]
